# YouTube Viewing and Content Quality in Toddlers

**DOI:** 10.1111/infa.70082

**Published:** 2026-03-19

**Authors:** Madalynn Woods, Maycee McClure, Alexandria Schaller, Heidi M. Weeks, Bolim Suh, Simran Chaudhry, Aimee Tibbitts, Heather Kirkorian, Rachel Barr, Sarah M. Coyne, Jenny Radesky

**Affiliations:** ^1^ Department of Pediatrics University of Michigan Medical School Ann Arbor Michigan USA; ^2^ Department of Nutritional Sciences University of Michigan School of Public Health Ann Arbor Michigan USA; ^3^ School of Human Ecology University of Wisconsin Madison Wisconsin USA; ^4^ Department of Psychology Georgetown University Washington DC USA; ^5^ School of Family Life Brigham Young University Provo Utah USA

## Abstract

YouTube is the most popular video‐sharing platform for young children and is largely characterized by low content quality. This study examined associations between YouTube viewing in toddlers, family demographics, child executive functioning (EF) and YouTube content quality. Participants include 361 largely white/non‐Hispanic (72%) parents and their 24‐to 26‐month‐olds (50% female) in a community‐based cohort study; data from the baseline wave is used in this analysis. Parents completed surveys and children completed three EF tasks (Snack Delay, Shape Stroop, Reverse Categorization task). Parents reported whether their child watched YouTube or YouTube Kids, and links to the last 10 videos viewed were collected. A total of 1032 videos were coded for 6 different features, and a total quality score was calculated for each video. YouTube viewing was very common: 258 (71.5%) toddlers watched YouTube or YouTube Kids versus 103 (28.5%) toddlers who never watched YouTube. YouTube viewing was associated with parent minoritized race/ethnicity, unemployment, single parenting, and higher child daily screen time. Videos had high levels of attention‐capturing “bedazzling” features (39.1%) and vicarious pleasure (48.6%), but fewer had high levels of educational content (16.7%) or positive role modeling (15.4%). Child EF scores were not associated with the content of YouTube videos viewed. Predictors of higher‐quality YouTube content viewing included higher income and children not attending childcare. These results have implications for both YouTube platform design and parent decision‐making about content.

## 1 Introduction

YouTube is the most popular video sharing platform among young children. In 2025, 84% of parents of children ages 2 to 4 and younger reported that their child ever watched Youtube and 51% watched YouTube daily (McClain et al. 2025), and some studies show that children view several hours of YouTube per day (J. S. Radesky, Weeks, et al. 2020; Mann et al. 2025). Young children are a key audience on YouTube, with many of its top‐viewed videos being nursery rhyme, learning‐oriented, or influencer channels directed to this age range (Gutelle 2025). Despite the major role this platform plays in the media diets of children globally, it remains relatively understudied. The current study was designed to help fill this gap by analyzing the content of YouTube videos viewed by toddlers and examining associations with child executive functioning and family characteristics.

As highlighted by the recent American Academy of Pediatrics guidelines about media and children (Munzer et al. 2026), children's digital media exposure goes beyond “screen time;” it is nested within multiple layers of individual differences, family context, and digital ecosystem design. To capture these multiple factors that shape early childhood media use, this study uses the Dynamic, Relational, Ecological Approach to Media Effects Research (DREAMER) conceptual model (Barr et al. 2024) as a theoretical framework. The DREAMER framework emphasizes the ways in which individual, family, and structural factors interact to shape the digital ecology in which children develop. For example, parents may choose media content with more attention‐capturing designs when children have lower self‐regulation, and the types of media parents choose will be determined in part by family resources (e.g., money, time) that dictate what media families can access and the degree to which parents can navigate a complex digital ecosystem to curate enriching digital experiences for their children. Therefore, in addition to examining associations of YouTube content design with child individual differences, the present study also examines how structural factors, including access to childcare and family resources, are related to YouTube family usage patterns.

### 1.1 Design Factors: YouTube Content Quality

Given the reach of YouTube to billions of children globally, it could be a vector for distribution of educational or prosocial media content. Yet, prior content analyses of videos watched by children on YouTube have revealed concerns about low‐quality content, a high amount of advertising and branding, and inappropriate depictions of violence or sexuality (Papadamou et al. 2020; J. S. Radesky, Schaller, et al. 2020). A 2025 content analysis by Bonus and colleagues found that only a third of YouTube videos contained education content, most of it of a shallow quality, and that 3‐quarters of children viewed at least one video with problematic content (Bonus et al. 2025).

Analysis of the most popular child‐directed channels has found that many contain extensive advertising, with some ads lasting longer than videos themselves (Yeo et al. 2021). This may be due to the recommendation feed, which drives 70% of views on YouTube (Solsman 2018) and, if trained on engagement metrics such as watch time, could prioritize more attention‐grabbing and emotionally arousing content (Cunningham et al. 2025), rather than prioritizing educational quality. Parents have reported that the automated feed of platforms like YouTube is hard for parents to monitor, since it introduces children to content and influencers parents feel their child is not ready for (J. Radesky et al. 2024).

Content quality is an important predictor of developmental outcomes during early childhood. Higher‐quality content is associated with stronger child language skills in meta‐analyses (Madigan et al. 2020). Although children who initiate TV viewing in infancy have weaker executive functioning (EF), watching high‐quality prosocial content protects against these risks (Nathanson et al. 2014). Research also shows that watching videos with higher educational content at a young age is protective against developing problematic media usage (Coyne et al. 2022). Given the high prevalence of YouTube usage, its financial incentives to earn ad revenue through engagement, and the power of its algorithmic feed to shape viewing habits, it is necessary to examine the quality of YouTube content to examine whether young children are offered content that supports their development or monetizes their time.

In response to criticisms about content quality, YouTube has released a series of guidelines and policies for creators of child‐directed videos in recent years (Age‐Appropriate Media for Kids and Teens, n.d.; Content Policies for YouTube Kids ‐ YouTube Help, n.d.). However, it is unclear whether young children's video offerings are improving on this platform and whether children are accessing higher‐quality content equitably. This study therefore aimed to conduct an in‐depth content analysis of more recently collected YouTube viewing histories (2022–2024), leveraging data from a community‐based study that measured children's self‐regulation and psychosocial context.

### 1.2 Individual, Family, and Structural Factors and YouTube Usage

The DREAMER framework emphasizes family and structural factors that shape children's digital media use. Prior work suggests that families with more socioeconomic adversity are more likely to use YouTube. One study of children ages 11 and under shows that YouTube viewing is more common in children from minoritized races/ethnicities and in families with lower educational attainment (Auxier et al. 2020). This may be because YouTube is a free, easily‐accessible media platform on most mobile devices and smart TVs, so families do not need to pay a monthly subscription to access child‐oriented content. Another study of 3‐ to 4‐year‐olds had similar findings regarding child race/ethnicity and parent education, but also found that children in center‐based child care programs were less likely to watch YouTube (Radesky et al. 2022). Consistent with the DREAMER model, this suggests that structural determinants of early childhood wellbeing, such as access to childcare or family experiences of stress and discrimination, may shape media practices. Only one prior study has examined whether YouTube video content quality is associated with child characteristics: Henderson et al. (2024) found that infants and children whose parents had lower educational attainment were more likely to watch age‐inappropriate YouTube videos.

Parent media use practices also strongly influence child media habits (Pyne et al. 2025) through shared family media routines, role modeling, and parents arranging for their children to use screens while parents use media themselves (Torres et al. 2021). Parents who use more mobile apps and technology may have a more positive attitude about apps like YouTube. Therefore, we aimed to investigate whether parents' daily smartphone habits predicted children's likelihood of YouTube usage.

The DREAMER model also posits that individual child characteristics such as self‐regulation and EF may also shape their media use habits in early childhood, either through parental motivations to regulate negative child behavior with screens, or through the child's preference for specific content and shows. Self regulation and EF have not been examined with respect to YouTube media habits. In early childhood, weaker attention regulation means that toddlers' attention may be more easily captured by high‐pleasure content with more salient formal features (e.g., visual and sound effects) (Courage and Setliff 2010), which YouTube then recommends more through its algorithmic feed. A Common Sense Media YouTube content analysis (J. S. Radesky, Schaller, et al. 2020), reported that many YouTube channels posted “wish fulfillment” content that displayed pleasurable activities such as eating candy, playing with toys, trying on princess costumes, or making slime—a unique genre to YouTube that does not appear on traditional TV programming. Children with weaker delay of gratification (the ability to wait to receive a reward) or inhibitory control (the ability to block a prepotent response) might be particularly drawn to this type of content or have difficulty resisting following attention‐grabbing feed recommendations. Additionally, children with weaker cognitive flexibility (the ability to switch between rules in your mind) may have more difficulty transitioning away from YouTube content, which could lead to longer daily screen time.

### 1.3 The Present Study

Together, prior research supports the DREAMER framework insofar as individual, family, and structural factors relate to children's screen use in general, and a small number of recent studies extend this to YouTube viewing. However, much remains to be tested, particular as it relates to video quality, not just quantity. Therefore, this study aimed to examine the following research questions about which toddlers watch YouTube, the content quality of videos they are watching, and whether YouTube media diet quality is shaped by child and family characteristics. We specifically focus on toddlers (2 years of age) in this study because it is when most US children are regularly engaging with screen media on a daily basis (McClain et al. 2025), there is a large amount of early childhood‐focused content on YouTube (e.g., nursery rhyme videos), and toddler media habits predict later trajectories of screen media use (McArthur et al. 2020). Further, this is a unique period of focus when it comes to media use given this is an age when children are beginning to comprehensive video narratives (Pempek et al. 2010) yet are still limited in their ability to transfer what they see in video to real‐life situations (Strouse and Samson 2020).

RQ 1 Which child and family characteristics were associated with toddlers' YouTube viewing?

H 1 Toddlers who watch YouTube will have weaker EF, will be more likely to be from lower‐income and lower‐education households, be less likely to attend childcare, and have parents who have longer daily smartphone use. We conducted exploratory analysis on other family characteristics (parental age, marital status, employment, and depression symptoms), for which we did not have a directional hypothesis.

RQ 2 What is the content quality of YouTube videos watched by toddlers?

H 2 Videos watched by toddlers will be more likely to contain attention‐capturing, branded, and vicarious pleasure content, compared to high educational quality.

RQ 3 Are child and family characteristics associated with the child's YouTube viewing quality?

H 3 Children with weaker EF will view lower quality YouTube content characterized by high levels of attention‐capturing, branded, and vicarious pleasure content. We will also examine whether family and household factors (income, parental education, age, marital status, employment, and depression symptoms) are associated with overall content quality, but do not hypothesize a directionality.

## 2 Methods

### 2.1 Overall Study Design

We analyzed data from the baseline wave of a community‐based cohort study of toddlers (24‐ to 26‐month‐olds) and their parents in a midwestern US state. Participants completed a study visit in their home or in the university research lab, consisting of surveys about family characteristics and a log of recent YouTube viewing history, direct measures of parent and child executive functioning (EF), and passive sensing of parental mobile devices. The study was approved by the [masked for review] IRB and was conducted in accordance with the ethical standards of the American Psychological Association. Families received up to $75 as a gift card or check for participating in the baseline wave.

### 2.2 Participants and Recruitment

We recruited 361 parents of toddlers via online advertisements, pediatrics clinic flyers, electronic medical record messages, word‐of‐mouth through the lab's community advisory board, and community partnerships (e.g., libraries, childcare centers). Sample size was determined based on anticipated attrition over 3 data collection waves, and power needed to detect small‐to‐moderate effect sizes in cross‐lagged structural equation models. Eligibility criteria included: (1) Parent was 18 years or older with physical custody and legal guardianship of a toddler between 24 and 26.99 months old, (2) target child had no major medical or developmental diagnoses, (3) the primary language spoken in the home was English, (4) the family lived within 50 miles of campus for a home or lab visit, and (5) to ensure that the enrolled parent could accurately report on the child's media usage habits, they needed to live with the index child at least 5 out of 7 days per week. The final sample included 361 pairs who provided written informed consent for themselves and their child.

### 2.3 Child YouTube Viewing

We assessed YouTube viewing through the question “Does your child watch YouTube?”. Response options were “No,” “Rarely/Occasionally,” and “Yes, regularly.” Parents who replied “Yes” also specified whether their child usually watched YouTube, YouTube Kids, or both. Almost half (175 [48.5%]) of participants watched YouTube regularly, 20 (5.5%) watched YouTube Kids regularly, 25 (6.9%) watched both regularly, and 38 (10.5%) used either platform rarely/occasionally. For regular YouTube viewers (*n* = 200), the research team assisted the family in collecting the most recent 10 links of YouTube videos viewed by the child. For the 38 occasional YouTube viewers, parents had the option of submitting video links, which were only submitted by 2 families. YouTube Kids‐only viewers did not submit viewing links; this was because history and links were not available on the app interface until February 2025, which was after baseline wave data collection was complete. Links were not collected for 42 regular YouTube viewers, because the child did not watch under an account, watch history was not turned on, the parent did not have access to the device where the child usually watched YouTube, or the parent chose not to provide links. When children watched YouTube under a parent's account, the examiner ensured that the parent's viewing history was not included. Two participants who submitted links had no codable links (videos were no longer available). At least 1 codable link was available for 158 participants, who watched a total of 1032 unique codable YouTube videos.

### 2.4 YouTube Content Coding

Each YouTube video link contains a unique video ID. We used Python code to scrape publicly available data for each video from the YouTube website, including video name, channel, upload date, and duration. We calculated video age (in years) by subtracting the upload date from June 1, 2025.

To develop a coding scheme, we adapted the Common Sense Media content coding approach (J. S. Radesky, Schaller, et al. 2020) for an early childhood audience. Investigators viewed hundreds of videos from the current sample, and met weekly to iteratively refine the coding scheme, and to decide upon final codes. For example, we started with a code for fast pacing but decided to eliminate it based on difficulty achieving consensus and reliability. Because content such as violence was rare in the sample, we instead developed codes for positive and negative role modeling, to better reflect what we felt would be more relevant to young children's informal learning from media characters (Holmgren et al. 2023). Final codes are summarized in Table 1 and encompass the formal features of the video, behavior of the characters, presence of branded products, and content (both educational and vicarious pleasure). Although YouTube contains high numbers of advertisements per video, ad frequency and type differ between users and usage patterns; therefore, reliable measures of ad counts were not possible. The full coding scheme is available in supplemental material.

**TABLE 1:** YouTube content codes, description, and prevalence in 1032 videos watched by toddlers.

Content code	Description	Median [IQR] or *n* (%)
Video length (min)	Data collected from YouTube.com for each video	12.1 [3.5, 45.1]
Years since upload	Years since date the video was uploaded to YouTube (calculated on June 1, 2025)	3.64 [2.18, 6.28]
Bedazzling (0/1)	Describes the formal features of the video—such as extraneous visual and sound effects, animations, or other features that might impose a cognitive load on a younger viewer or distract during viewing. It includes “filler” features like giggles and animations on the screen seemingly designed to capture attention.	0: 629 (61.0%)
		1: 403 (39.1%)
Positive role modeling^a^ (0/1/2)	Character shows discrete, observable pro‐social or healthy behavior such as empathy, perspective taking, cooperation/helping, being flexible, caring for animals or sick people, calming down emotions, handling frustration, wearing sunscreen, eating healthy foods, brushing teeth, or safety. This can include showing negative behavior initially but meaningfully resolving it (such as apologizing, trying to make things better). Videos score a 1 for brief or low‐degree positive behaviors, such as saying please/thank you or doing something brave. A video receiving a 2 has more overt positive content such as healthy eating or lessons about emotions.	0: 456 (47.8%)
		1: 352 (36.9%)
		2: 147 (15.4%)
		(77 not codable)
Negative role modeling^a^ (0/1/2)	Character shows negative, impulsive, violent, rude or unsafe behavior or exhibits over‐focus on appearance, competition, or consumption. Includes any unsafe behavior that a child might be tempted to do, without a warning like “make sure a grown up does this.” Videos score a 1 for low‐degree negative behavior, such as vanity or brief unsafe behaviors. A score of 2 indicates more severe negative behaviors such as violence.	0: 747 (78.1%)
		1: 155 (16.2%)
		2: 54 (5.7%)
		(76 not codable)
Branded content (0/1)	Featuring or intentional placement of branded toys, food, candy, clothes, cars/vehicles, hotels, museums/destinations, etc. Character might say the brand name out loud (“Here's my Lambo”), call explicit attention to the brand (“I'm using X brand markers”), or the video takes place in a branded location (e.g., store, aquarium).	0: 868 (84.1%)
		1: 164 (15.9%)
Vicarious pleasure (0/1)	Video shows pleasurable activities ‐ such as eating candy, playing with toys, making extravagant messes, showing off luxury items, wearing expensive outfits, or taking part in activities that kids might wish to do (e.g., go karts, having a birthday party, finding buried treasure, playing video games). Could also include satisfying content such as “calm your baby down” sensory animations, slime videos, chocolate pouring, or speed‐painting.	0: 531 (51.5%)
		1: 501 (48.6%)
Educational content (0/1/2)	Describes the educational quality of videos, taking into account both the depth of curricular content and how it is delivered. For example, a video might have a lot of different labels or ideas in it, but if it is delivered in a fast pace or shallow manner, it would be a 1. In order to code a 1 or 2, the coder needs to be able to identify a learning goal (e.g., pre‐academic skills, social‐emotional learning) that is delivered according to how toddlers learn (e.g., repetition, showing in different contexts, tying to child's experience, ask questions and pause, etc.) A video that is given a 2 may demonstrate the presence of a curricular goal, re‐teaching of a concept in several ways, call and response with the viewer, and/or presenting the idea in a way that a young child could apply in their real life. A video scored a 1 may simply demonstrate the presence of a curricular goal, such as characters reciting colors or numbers.	0: 481 (50.4%)
		1: 315 (33.0%)
		2: 159 (16.7%)
		(77 not codable)

^a^ Positive and Negative Role Modeling codes are not mutually exclusive.

We trained 9 coders to reliability (*κ* = 0.78–1.0). Each coder watched each video on a logged‐out Chrome browser for a minimum of 10 min. For videos in a non‐English language, coders gave scores for Bedazzling (e.g., superficial visual enhancements), Branded Content, and Vicarious Pleasure, as these codes are generally visible without language. Since role modeling and educational content require understanding of the language spoken, coders marked these variables as uncodable unless content was clearly seen, such as violence, or were videos easily found in English, such as popular songs from movies. Coders met monthly to resolve coding uncertainties by consensus. Coders marked whether the video was “Made for Kids” based on the appearance of a prompt under the video to watch it on YouTube Kids, and a lack of comments section.

### 2.5 Parent and Child Characteristics

#### 2.5.1 Family Demographics and Psychosocial Context

Parents completed surveys on REDCap (Harris et al. 2009, 2019) including their age, race/ethnicity (options included White, Black, Hispanic, Middle Eastern/North African, American Indian/Alaska Native, East Asian, South Asian, Southeast Asian, Native Hawaiian/Pacific Islander, and Multiple Races; collapsed into white non‐Hispanic vs. minoritized because of small cell sizes for most groups), educational attainment (options included did not graduate from high school, high school, GED, some college courses, 2 years college degree, 4 years college degree, and more than 4 years college degree; collapsed into 2‐year degree or less, 4‐year degree, and more than 4‐year degree), marital status (options included single, never married, in a committed relationship with a partner, married, separated or divorced, widowed; collapsed into single/divorced vs. married/partner), employment status (options included unemployed and not currently seeking employment, unemployed and seeking employment, one part‐time job, one full‐time job, and multiple jobs; collapsed into unemployed vs. employed), household size and income (from which we calculated the income‐to‐needs ratio), and their child's sex. Parents reported depression symptoms on the Center for Epidemiologic Studies Depression Scale (CES‐D) (Radloff 1977), a widely used validated scale that asks parents to rate the presence of depression symptoms (e.g., “I felt depressed,” “I could not get going”) over the past week on a 0 to 3 scale (Cronbach's alpha = 0.88).

#### 2.5.2 Parent Smartphone Usage

At the study visit, parent smartphone usage was assessed through collection of iPhone Screen Time screenshots or installation of the passive sensing app, Chronicle, on Android smartphones for 9 days. Screenshots captured the average daily minutes of iPhone usage for the past 7 days, and individual app usage was manually entered into Excel files for cleaning and analysis. The Chronicle app provided timestamped output of app usage, which was analyzed after cleaning (i.e., inspection for data gaps or long‐running apps; removal of first and last partial days). Average daily smartphone usage duration was calculated after removal of apps that were likely child usage. There were 259 parents with iPhones (229 with average daily duration data) and 102 parents with Android devices (88 with average daily duration data). Average daily duration data was missing for 44 parents because they did not submit screenshots, submitted incorrect or incomplete screenshots, did not have Screen Time turned on, did not install Chronicle on their phone, or had technical difficulties.

#### 2.5.3 Child Screen Media Duration

Child screen time was assessed with the questions: “Thinking about a typical weekday (Monday–Friday)/weekend (Saturday–Sunday), how much time does your child spend watching videos or programs on a TV (either through network TV, cable, or smart TV apps)?” with the following response options: (Never, Less than 30 min, 30 min to 1 h, 1–2 h, 2–3 h, 3–4 h, 4–5 h, More than 5 h). A continuous value for daily screen time was calculated by assigning each child the midpoint value from the response category and creating a weighted average of weekday and weekend values. Although this question specified viewing media on a TV, and therefore omitted sources of screen time from other devices, 79% (*n* = 286) of participants were asked where their child mostly watches TV programs/videos; 72.4% mostly watched videos on a TV set, 12.9% mostly on a mobile device, and 14.7% on both. This subset of participants was also asked about their child's TV/video viewing on any device, and there was a strong correlation between TV/video watching on a TV and on any device (Spearman *r* = 0.87, *p* < 0.001). Due to this, we chose to use responses from the child screen time question that all participants in the sample were asked.

#### 2.5.4 Child Executive Functioning

We characterized toddler EF (delay of gratification [DoG], inhibitory control [IC], and cognitive flexibility [CF]) using three tasks. These tasks capture both hot (emotion regulation, DoG task) and cool (cognitive, IC and CF tasks) EF (Zelazo and Carlson 2012) to test whether different EF functions are associated with YouTube viewing.

In the Snack Delay (DoG) task (Kochanska et al. 2000), a sweet snack was placed under a clear cup and children were instructed to take the snack from under the cup after a bell was rung. There were a total of 4 trials of increasing duration; 5, 10, 15, and 20 s delays. Halfway through each trial, the researcher lifted their hand as if they were about to ring the bell to demonstrate a “fake out”, then reset before ringing the bell after the delay period ended. Scores are recorded on each trial, quantified as 0 = child eats the snack immediately or before the “fake out”, 1 = child eats the snack during the “fake out”, 2 = child reaches for or touches the cup, snack, bell, or timer before the bell is rung, and 4 = child waits for the bell to ring before taking the snack. Scores are averaged across the 4 trials, with higher scores indicating stronger DoG.

In the Shape Stroop (IC) task (Carlson et al. 2004), after confirming that children could identify the different fruits (apple, orange, banana), children were presented with 3 cards with little fruits embedded in a larger picture of a different fruit. Children were asked “point to the little [fruit],” which required them to inhibit a prepotent response to point to the larger picture of the fruit in front of them. There are a total of 3 trials with the child pointing to the little apple, the little orange, and the little banana. Scores for each trial were assigned as 0 = child points to incorrect fruit, 1 = child points to incorrect fruit, then self‐corrects to correct little fruit, and 2 = child points to correct little fruit. Scores were averaged over the 3 trials.

Lastly, children completed the Minnesota Executive Function Scale (MEFS) reverse categorization task. This validated tablet‐based assessment first asked children to sort objects by size, by dragging and dropping objects into the corresponding size box, but then the rule changed and children were asked to drag and drop objects into the opposite size box. If children passed this level, they then were asked to sort objects by color and shape. Cognitive flexibility was indexed by children's ability to learn the new rule and to switch their response. Age‐ and sex‐adjusted standard scores (mean 100, SD 15) were based on both accuracy and response time.

### 2.6 Data Analysis

*RQ1*: We calculated the frequency of parent‐reported YouTube viewing (regular YouTube viewer, regular YouTube Kids viewer, regular viewer of both, occasional use of either, no use of either YouTube or YouTube Kids) and collapsed this into a binary variable—ever or never watching YouTube/YouTube Kids (described hereafter as “YouTube viewer;” we justified collapsing YouTube and YouTube Kids viewers because > 70% of participants viewed Made For Kids videos, which are available on both platforms). We conducted Chi‐Square tests, *t*‐tests (for parent age), and Wilcoxon Two‐Sample tests to examine bivariate associations between YouTube viewing and child and family characteristics. To examine independent predictors of YouTube viewing, we then built a multivariable logistic regression model using Firth bias‐correction and included all child and family characteristics that had a *p‐*value of < 0.20 in bivariate tests.

*RQ2*: We conducted descriptive statistics for all content codes, video duration, and years since upload. To examine whether content codes tended to co‐occur, or correlated with the age of video, we calculated Kendall's tau‐b correlations (as coded data had levels of 0/1 or 0/1/2) between all video codes.

*RQ3*: We calculated a total content quality score for each video (sum(Positive Role Modeling, Educational Content) ‐ sum(Bedazzling, Negative Role Modeling, Branded Content, Vicarious Pleasure)). In the subsample of 158 children with codable YouTube links, we calculated an average score for each content code and an average total content quality score, based on the videos the child watched. If a child watched a specific video more than once, it was counted multiple times when calculating the average scores.

To assess whether child EF predicted the content quality of videos watched, we conducted Spearman correlations between Snack Delay score (DoG), Shape Stroop score (IC), and MEFS standard score (CF) individually with children's average scores for select content codes (Bedazzling, Branded Content, and Vicarious Pleasure). Total content quality scores were normally distributed but other continuous variables were skewed, so we conducted *t*‐tests and Spearman correlations to examine bivariate associations between children's average total content quality scores and child and family characteristics, including child EF. To examine independent predictors of children's average total content quality scores, we then built a multivariable linear regression model including all child and family characteristics that had a *p‐*value of < 0.20 in bivariate tests.

## 3 Results

### 3.1 Participant Characteristics

In the full sample (Table 2), parents were primarily mothers (93.1%), identified as white non‐Hispanic (72.1%), with an average age of 34.6 years; 93.3% were married or lived with a partner, 52.8% had more than a 4‐year college degree, and 22.8% were unemployed or stayed at home. Median daily parent smartphone duration was 5.3 h, ranging from 1.4 to 16.2 h/day. Children were 50% female, 47.9% were enrolled in childcare, and median daily screen duration was about 1 h, ranging 0–5.5 h/day.

**TABLE 2:** YouTube usage among toddlers and associations with child and family characteristics.

Characteristic	Overall (*n* = 361) Mean (SD) Median [IQR] or *n* (%)	YouTube users (*n* = 258) Mean (SD) Median [IQR] or *n* (%)	YouTube non‐users (*n* = 103) Mean (SD) Median [IQR] or *n* (%)	Bivariate test^a^ *p*‐value	aOR^b^ (95% CI) for YouTube usage
Child sex female	177 (50.0%)	122 (48.2%)	55 (54.5%)	0.29	—
Child attends childcare (in‐home or center‐based)	170 (47.9%)	119 (47.0%)	51 (50.0%)	0.61	—
Child snack delay score	3.5 [2.0, 4.0]	3.5 [2.0, 4.0]	4.0 [2.0, 4.0]	0.12	1.09 (0.89, 1.33)
Child shape stroop score	0.33 [0, 0.67]	0 [0, 0.67]	0.33 [0, 0.67]	0.65	—
Child MEFS standard score	101 [94, 105]	101.0 [94.0, 105.0]	99.0 [94.0, 105.0]	0.87	—
Child daily TV/video viewing (h)	0.96 [0.39, 1.79]	1.29 [0.61, 1.79]	0.61 [0.18, 1.50]	< 0.001	1.37 (0.998, 1.87)
Parent age (y)	34.6 (4.8)	34.6 (5.0)	34.7 (4.3)	0.88	—
Parent married/partner	332 (93.3%)	230 (90.6%)	102 (100%)	0.001	0.05 (0.003, 0.88)
Parent minoritized race/ethnicity	99 (27.9%)	87 (34.3%)	12 (11.9%)	< 0.001	2.48 (1.13, 5.42)
Parent education				0.024	
2‐year degree or less	61 (17.1%)	52 (20.5%)	9 (8.8%)		1.43 (0.46, 4.38)
4‐year degree	107 (30.1%)	76 (29.9%)	31 (30.4%)		1.03 (0.54, 1.96)
> 4‐year degree	188 (52.8%)	126 (49.6%)	62 (60.8%)		Ref
Parent not employed/stays at home	80 (22.8%)	64 (25.7%)	16 (15.7%)	0.042	2.48 (1.15, 5.38)
Parent CES‐D score	7.0 [3.0, 12.0]	8.0 [4.0, 13.0]	5.0 [3.0, 9.0]	0.004	1.04 (0.995, 1.09)
Parent average daily smartphone use (h)	5.3 [3.9, 7.2]	5.4 [4.0, 7.3]	4.9 [3.4, 6.7]	0.12	0.94 (0.83, 1.07)
Household ITN	4.01 [2.83, 4.84]	4.01 [2.61, 4.84]	4.10 [3.26, 5.00]	0.066	1.19 (0.94, 1.52)

^a^ *t*‐test, Wilcoxon Two‐Sample test, or Chi‐Square test.

^b^ Adjusted for all other listed variables with a bivariate *p* value < 0.20.

### 3.2 RQ1: Associations of Child and Family Characteristics With YouTube Viewing

YouTube viewing was very common, occurring in 71.5% of the sample. Overall, 103 (28.5%) children never watched YouTube.

As shown in Table 2, in bivariate comparisons, children were more likely to watch YouTube if they had longer daily screen time or if they had a parent who was single/divorced, identified as minoritized race/ethnicity, had lower educational attainment, was not employed/stayed at home, or had higher depression symptoms. In multivariable models, YouTube usage was less common in children whose parent was married/had a partner versus single/divorced parents (aOR = 0.05 [95% CI: 0.003, 0.88], *p* = 0.041). YouTube usage was more common if the parent identified as minoritized race/ethnicity compared to white non‐Hispanic parents (aOR = 2.48 [1.13, 5.42], *p* = 0.023), and in children whose parent stayed home or was unemployed versus parents with part‐ or full‐time jobs (aOR = 2.48 [1.15, 5.38], *p* = 0.021). Marginal associations were found for YouTube usage and longer daily screen time (aOR per hour of screen duration/day = 1.37 [0.998, 1.87], *p* = 0.052).

### 3.3 RQ2: YouTube Content Quality

Of the sample of 1032 codable YouTube videos viewed by participants, 97 were in a non‐English language. The most common video genres were songs (324 videos), programs created for TV or another platform (e.g., Disney, Netflix, Sesame Street; 227 videos), and programs created for YouTube (e.g., Blippi, Miss Rachel, 222 videos). The majority of videos viewed (763 [74%]) had a Made For Kids label, indicating that the creator intended the video to be viewed by children. About one‐third of videos (334) were compilations that combined multiple songs or episodes from the same show. Median video duration was approximately 12 min, ranging from 0.1 to 715 min. Frequencies of content codes are shown in Table 1. Attention‐capturing “Bedazzling” features occurred in 403 videos (39.1%), such as glowing or flashing visual elements or extraneous giggling. Brief or low degrees of Positive Role Modeling (e.g., Bluey wearing her seatbelt) occurred in 352 (36.9%) videos, and stronger, more prevalent Positive Role Modeling (e.g., characters sharing and discussing fairness), occurred in 147 (15.4%). Negative Role Modeling was relatively less common, occurring briefly in 155 (16.2%) videos, and more pervasively (e.g., continuous mocking or teasing), in 54 (5.7%). Branded Content such as toys, name‐brand food/candy, clothing, or specific locations (e.g., Blippi videos with explicitly named zoos or aquariums) were present in 164 (15.9%) videos.

Vicarious Pleasure was a common theme in many videos (501 [48.6%]), consisting of satisfying videos like watching construction trucks drive around, planning an extravagant birthday party, going on a treasure hunt, or having the perfect day at the beach.

Shallow or simple Educational Content was present in 315 (33.0%) videos, while 159 (16.7%) videos contained higher Educational content. When we examined correlations between video features and content codes (see Table 3), we found that newer videos tended to be longer in duration and have more attention‐capturing features such as Bedazzling, Branded Content, and Vicarious Pleasure (which themselves were moderately inter‐correlated), but also had higher Educational and Positive Role Modeling scores. Videos with higher Educational scores tended to be longer and have more Positive Role Modeling and less Negative Role Modeling, but also had weak positive correlations with Branded Content and Bedazzling.

**TABLE 3:** YouTube content code inter‐correlations^a^ in 1032 videos watched by toddlers.

Video code	Video length	Years since upload	Bedazzling	Positive role modeling	Negative role modeling	Branded content	Vicarious pleasure
Video length	1						
Years since upload	−0.19	1					
	< 0.001						
Bedazzling (0/1)	0.10	−0.22	1				
	< 0.001	< 0.001					
Positive role modeling (0/1/2)	0.23	−0.10	−0.02	1			
	< 0.001	< 0.001	0.55				
Negative role modeling (0/1/2)	0.01	−0.06	0.12	−0.05	1		
	0.65	0.013	< 0.001	0.094			
Branded content (0/1)	0.19	−0.09	0.27	−0.03	0.18	1	
	< 0.001	< 0.001	< 0.001	0.30	< 0.001		
Vicarious pleasure (0/1)	0.21	−0.18	0.30	−0.005	0.13	0.33	1
	< 0.001	< 0.001	< 0.001	0.88	< 0.001	< 0.001	
Educational content (0/1/2)	0.30	−0.14	0.09	0.23	−0.16	0.08	−0.06
	< 0.001	< 0.001	0.003	< 0.001	< 0.001	0.006	0.053

^a^ Kendall's tau‐b correlations and *p*‐values reported for all variables.

### 3.4 RQ3: Predictors of Watching Higher‐Quality YouTube Videos

Contrary to our hypotheses, children's EF (DoG, IC, and CF) was not significantly associated with the average scores of Bedazzling, Branded Content, or Vicarious Pleasure in the YouTube videos they viewed (all *p*‐values > 0.05, data not shown).

Across all videos for total content quality could be calculated (i.e., all 6 codes were available), the average score was 0.03 (SD 1.78), ranging from −5 to 4.

In the 158 children with codable YouTube links, the average child‐level total content quality score was 0.28 (SD 1.32), ranging −2.7 to 3.5. As shown in Table 4, total content quality scores were significantly higher in children who did not attend child care (adjusted *B* = 0.64 [95% CI: 0.17, 1.11], *p* = 0.008) and those with higher income (adjusted *B* = 0.17 [0.02, 0.33], *p* = 0.026) in multivariable models. There were no associations between child EF and total content quality scores. In post‐hoc analyses, we found that toddlers who could not complete any trials in the Snack Delay task, and were therefore missing a score, were more likely to be YouTube viewers (89% vs. 70%, *X* ^*2*^ *(1) = 5.56*, *p* = 0.018).

**TABLE 4:** Associations of participant characteristics with total content quality score in 158 children with coded YouTube links.

Characteristic	Sample (*n* = 158) Mean (SD) Median [IQR] or *n* (%)	Total content quality score Mean (SD) by category or spearman r	Bivariate test *p*‐value^a^	B^b^ (95% CI)
Child sex			0.12	
Female	75 (48.4%)	0.10 (1.33)		−0.29 (−0.74, 0.17)
Male	80 (51.6%)	0.43 (1.28)		Ref
Child attends childcare			0.13	
Yes (in‐home or center‐based)	68 (43.8%)	0.10 (1.20)		Ref
No	87 (56.1%)	0.42 (1.38)		0.64 (0.17, 1.11)
Child snack delay score	3.5 [2.0, 4.0]	−0.016	0.85	—
Child shape stroop score	0.50 [0, 0.67]	−0.11	0.22	—
Child MEFS standard score	101 [94, 105]	−0.12	0.19	−0.01 (−0.04, 0.01)
Child daily TV/video viewing (hours)	1.25 [0.61, 1.79]	−0.06	0.48	—
Parent age (years)	34.5 (5.1)	−0.13	0.10	−0.03 (−0.08, 0.02)
Parent marital status			0.37	
Married/partner	142 (91.0%)	0.30 (1.29)		—
Single/divorced	14 (9.0%)	−0.03 (1.52)		
Parent race/ethnicity			0.11	
Minoritized	62 (40.0%)	0.06 (1.45)		−0.16 (−0.62, 0.30)
White non‐Hispanic	93 (60.0%)	0.41 (1.21)		Ref
Parent education			0.28	
2‐year degree or less	31 (19.9%)	0.14 (1.53)		—
4‐year degree	48 (30.8%)	0.52 (1.34)		
> 4‐year degree	77 (49.4%)	0.17 (1.19)		
Parent employment status			0.48	
Not employed/stays at home	40 (26.1%)	0.40 (1.55)		—
Employed (part or full‐time)	113 (73.9%)	0.23 (1.24)		
Parent CES‐D score	7.0 [3.0, 13.0]	−0.06	0.47	—
Parent average daily smartphone use (hours)	5.3 [4.0, 7.5]	−0.05	0.57	—
Household ITN	4.00 [2.34, 4.84]	0.11	0.16	0.17 (0.02, 0.33)

^a^ *t*‐test, ANOVA, or Spearman r.

^b^ Adjusted for all other listed variables with a bivariate *p* value < 0.20.

## 4 Discussion

In this cross‐sectional, community‐based study of early childhood media use, we found high rates of YouTube usage in 2‐year‐olds, with half of the sample watching YouTube regularly. This is similar to 2025 estimates from the Pew Internet survey (McClain et al. 2025) and supports prior work showing that watching the main YouTube platform is much more common than using YouTube Kids in young children. Reasons for usage of the main YouTube platform may include ease for families, as it may already be available on smart TVs or mobile devices, rather than creating a YouTube Kids profile or installing a separate YouTube Kids app. Anecdotally, many of the parents in our study were not aware of YouTube Kids. Yet, viewing on the main platform opens children up to inappropriate content and data collection from Not Made For Kids videos.

YouTube viewers were more likely to have longer daily screen time—over double the median value of non‐viewers, so it is possible that YouTube contributes a significant amount of duration to daily viewing. With design affordances such as autoplay, recommendation feeds, livestreams of nursery rhymes, mobile device accessibility, and many videos of an hour or longer, it is plausible that YouTube takes up a longer proportion of a child's day than other screen experiences. It is also possible that families with more YouTube use have longer or no screen time limits.

### 4.1 Predictors of YouTube Viewing

YouTube usage was also more common in children whose parents identified themselves as minoritized race/ethnicity or were single/divorced, and marginally more common in parents with higher depression symptoms. Altogether, these family characteristics may signal less psychosocial support and YouTube may be a convenient way to provide engaging child‐directed content that keeps toddlers occupied. Indeed, occupying children is a relatively common reason parents give for their toddlers' screen time (Suh et al. 2024). These findings are consistent with predictions about the role of structural factors in influencing parent wellbeing and motivations for child media use, as described in the DREAMER Model. In stressed or marginalized families, effective strategies to improve early childhood media use could include shifting to more positive and prosocial media content, but we also hypothesize that it may be important to provide additional parenting or community resources.

Children of parents who were unemployed and presumably staying at home with their toddler also were more likely to use YouTube. Parents who are home with toddlers may struggle to find activities to keep toddlers busy, or may feel a greater need for a break from caregiving (Torres et al. 2021) through child media use. Unlike a prior study in 3–5‐year‐olds (Radesky et al. 2022), child care access was not associated with YouTube usage in this cohort. This difference may be due to different age ranges (i.e., more toddlers are at home with parents at this age) or the fact that the aforementioned study was conducted before the COVID‐19 pandemic, during which time both media practices and child care access changed (Piper et al. 2025).

YouTube usage was not associated with child executive functioning, for which there are several possible explanations. First, YouTube usage is common enough that child behavior or self‐regulation may not be a strong driver of this media practice. Second, EF is challenging to measure in 2‐year‐olds and overlaps considerably with temperament traits such as effortful control (Kim et al. 2013). In fact, many toddlers in our cohort could not finish the EF tasks due to behavioral disengagement. Since low‐quality content may influence early childhood behavior and the development of language and attention skills (Madigan et al. 2020; Nathanson et al. 2014), future longitudinal research should examine links between YouTube viewing and EF over time.

### 4.2 Content Quality Findings

Compared to content analyses performed on older data (J. S. Radesky, Schaller, et al. 2020), the quality of YouTube videos continued to be rather low in this cohort ‐ with high levels of attention‐capturing designs and content, and high‐level educational approaches in only ∼17% of videos and high levels of positive role modeling in ∼15% of videos. Notably, this is for videos actually viewed by toddlers and shared by parents in our study, rather than a random sample of all videos on YouTube. This may be because YouTube's ad‐based business model and algorithms trained to optimize watch time may prioritize more attention‐grabbing rather than prosocial or educational content. Indeed, we found that over time, videos with a more recent upload date had higher levels of attention‐capturing features: Bedazzling, Branded Content, and Vicarious Pleasure, which further supports the hypothesis that creators may be competing for attention and views.

These results are also similar to a more recent content analysis that found low levels of in‐depth educational content (Bonus et al. 2025). Interestingly, while deep educational content was lacking, parents perceived YouTube as a helpful tool for “interest‐based” learning, suggesting that non‐curricular videos may still be a valuable learning tool (Bonus et al. 2025).

Interestingly, Educational content correlated to a small degree with Bedazzling and Branded content, suggesting that some creators use these engagement tactics in learning‐oriented videos. It is also possible that creators are putting “surface level” educational material—such as simple naming of letters, numbers, or colors—into their videos so that they can tag them as “educational.” Although audio and visual effects can provide on‐screen learning support to guide toddlers' attention to relevant information (Wass and Smith 2015), the Bedazzling code specifically identified irrelevant enhancements that could distract from learning.

### 4.3 Predictors of Content Quality

Contrary to our hypotheses, child EF did not correlate with the content of YouTube videos they watched in terms of attention‐capturing or satisfying features. However, we only coded 10 of the child's recently viewed videos, which may not be a precise indicator of their overall viewing habits. Based on our data collection method, we were not able to assess whether children watched the whole video versus only a portion of it before skipping to another video. Additionally, at 2 years of age, parents may hold more control over what children watch on YouTube, which may change as children age, develop specific media preferences, and can control smart TVs and tablets through verbal commands.

The main family characteristics that positively predicted children's overall YouTube viewing quality were higher income and not attending childcare. The reason for a link with income is unclear, but could be due to overall family stress and parents' bandwidth to monitor content quality. Higher‐income parents may curate their child's media experiences more intensely (Livingstone and Blum‐Ross 2020). Since most children watched YouTube on their parents' account, algorithmic profiling based on parent data is also possible.

It is possible that parents whose children did not attend child care had an educational motivation to seek out higher‐quality educational videos and similarly had more time to monitor content. Child care access in the U.S. is limited; 28% of children in the US who need a child care spot cannot get one (Varisco 2026). It may be that parents who are impacted by this gap intentionally choose educational media to help supplement their child's learning.

As this research is cross‐sectional, future studies are needed that examine how content quality associates bidirectionally with the development of language, social‐emotional skills, and EF over time. A key mechanism worth examining in future research is how longer viewing times contribute to displacement of other activities.

### 4.4 Limitations

Families in our sample were of relatively high socioeconomic status, which limits the generalization of our findings. We found that socioeconomic status was negatively associated with rates of YouTube viewing and positively related to video content quality, so our results may underestimate viewing rates and overestimate content quality in the greater population. More research is needed with families of low socioeconomic status to fully understand the factors that contribute to YouTube viewing and YouTube content quality, as well as how various psychosocial factors, such as childcare use and employment rates, factor into these relationships. Additionally, the exclusion of families whose home language is not English also limits the generalizability of our results since almost 1 in 5 U.S. families speak another language at home (Dietrich and Hernandez 2022).

Our data collection was further limited by how YouTube links were collected. Not all families who reported regular YouTube viewing submitted video links, and distinguishing between regular and occasional YouTube viewing relied on parent subjective reports. We also were unable to collect links from families who only used YouTube Kids, which may bias our content quality findings. Future studies should expand surveying to obtain quantifiable data on how often YouTube is viewed by itself, separate from TV or other streaming platforms.

Furthermore, there are other content codes that may be relevant to children's executive functioning outcomes that were not captured in this present study. For example, other researchers like Henderson et al. (2024) have examined the pacing of video content, which may have a greater impact on children's attention. While we aimed to code each content feature independently of each other, other researchers like Neumann and Herodotou (2020) calculated educational content scores by considering how design features, like bedazzling or age appropriateness, obscures and reduces the educational content quality of the video. This holistic approach to content coding may shed additional insights on the overall quality of children's programming on YouTube, especially in light of YouTube's novel content genres, such as satisfying videos or video game livestreams. Finally, distracting advertising and short‐form videos have relevance for young children's attention regulation, but were not examined in this study.

## 5 Conclusion

Supporting healthy consumption of media in early childhood requires more than simple screen time limits (Munzer et al. 2026); factors related to the design of media platforms and family stressors also need to be addressed. Our current findings reveal that YouTube continues to contain substantial amounts of low‐quality content for 2‐year‐olds. Though the relationship between executive functioning and children's content quality on YouTube is not yet clear, parents who are concerned about negative role modeling, commercialism, or shallow learning in YouTube videos could opt for higher‐quality channels, such as Sesame Street or Ms. Rachel, which received some of the best scores in this sample. Concerns about content in children's programming can also be addressed by using alternative platforms, including non‐profit platforms such as PBS KIDS, which may serve as a higher‐quality alternative without commercial content.

## Author Contributions

**Madalynn Woods:** data curation, methodology, writing – original draft. **Maycee McClure:** data curation, methodology, writing – review and editing. **Alexandria Schaller:** data curation, methodology, project administration, writing – review and editing. **Heidi M. Weeks:** data curation, methodology, writing – review and editing. **Bolim Suh:** data curation, investigation, writing – review and editing. **Simran Chaudhry:** data curation, methodology: writing – review and editing. **Aimee Tibbitts:** data curation, methodology, writing – review and editing. **Heather Kirkorian:** conceptualization, data curation, funding acquisition, methodology, writing – review and editing. **Rachel Barr:** conceptualization, funding acquisition, methodology, writing – review and editing. **Sarah M. Coyne:** conceptualization, funding acquisition, writing – review and editing. **Jenny Radesky:** conceptualization, funding acquisition, methodology, project administration, formal analysis, writing – original draft.

## Funding

This study was funded by the Eunice Kennedy Shriver National Institute of Child Health and Development (Grants R01HD102370 and P01 HD109907). REDCap and recruitment support was provided through the Michigan Institute for Clinical & Health Research (Grant CTSA: UM1TR004404).

## Ethics Statement

APA ethical standards were followed in the conduct of the study.

## Conflicts of Interest

The authors declare no conflicts of interest.

## Supporting information


Supporting Information S1

## Data Availability

Data available upon reasonable request.
