# Supplementary material for: YouTube Viewing and Content Quality in Toddlers
Source: Infancy. 2026 Mar 19;31(2):e70082. doi: 10.1111/infa.70082 (PMC13002997; doi:10.1111/infa.70082)
Supplement: Supplementary file 1 — Supporting Information S1 [file INFA-31-0-s001.docx]

**YouTube Content Coding**

**Description:** The goal of this coding scheme is to assess the content quality, advertising, and design of YouTube videos watched by children in the P01 Growing up in a Digital World studies (UM MITTen, UW/Georgetown EMU, BYU Project MEDIA). The coding approach will assume that viewers are young children (5 years old or younger) when determining age-appropriateness. (See bottom of this coding scheme for information on what makes videos educational for toddlers and young children.)

**Coder instructions:**

- Watch the first **10 minutes** of each video, it is ok to watch at 1.5x or 2x speed as long as you are able to discern Bedazzling and other codes.
- If video is longer than 10 minutes, advance intermittently at 5 minute intervals and watch for about 30 seconds. This will let you capture any interstitial ads and any neg/pos role modeling or branded content.
- Advance to the end of the video and watch for any end-roll ads
- If there is a compilation where the content seems to change a lot, and you are not sure how to code, bring to group to discuss.
- Code while logged out of a Chrome browser, not incognito mode, browser tab maximized, NOT in theater mode or with video full-screen (so you can see what ads appear underneath the video or in the sidebar)
- Turn off auto-play

—----------------------------------------------------------------------------------------------------------------------------------------------------------------------------------------

**GENERAL CODES**:

**Language:** Language spoken or written in text in the video (not in the title).

- English vs other
- If the video itself does not have any words, code based on the title of the video

For non-English videos, code the following as missing:

- Pos_role_modeling (code 999)
- Neg_role_modeling (code 999)
- Educational content (code 999)

**Genre:**

- **Show (YT)** - Show that was made for YouTube e.g., Blippi, Mother Goose Club
- **Show (TV)** – Show that was originally made for TV/streaming service: e.g., Bubble Guppies, Sesame Street, Baby Einstein, Paw Patrol, Daniel Tiger. You may need to google this to find out.
- **Movie** (clips/trailer/TV trailer - including movie songs eg from Moana)
- **Songs** – includes kid songs, nursery rhymes
- **Music video** (e.g., by a pop star or recording artist)
- **Influencer** (e.g., Ryan’s toy review, Kids Diana Show, Vlad + Niki, Like Nastya, FGTeeV)
- **Toys** - unboxing, reviews, playing with (but not done by influencers)
- **Info** (Instructional/DIY/information - e.g., facts about dinosaurs, how to cook or repair something)
- **Satisfying** (- e.g., slime, videos of trains or construction vehicles without any voiceover teaching about trains)
- **Arts/sports** (e.g., culture - video of a dance recital, ballet or street performance)
- **Video gaming** (shows someone playing video games)
- **Humor**
- **Book Readalong**

**Compilation:** yes(1) / no(0) Videos count as compilations if the YouTuber/content creator made the compilation by splicing together other content, or states that it is a compilation in the description/title.

**Made_for_kids:** (0 vs 1) Code 1 if below the video there is a symbol showing YouTube Kids

—----------------------------------------------------------------------------------------------------------------------------------------------------------------------------------------

**CONTENT QUALITY**:

**Bedazzling:** (0 vs 1) This code describes the formal features of the video – such as extraneous visual and sound effects, animations, or other features that might impose a cognitive load on a younger viewer or distract during viewing. It does not apply to background music in song videos or the actions of the characters - only if there are extraneous visual or sound effects on top of these features. It also captures the phenomenon of YouTubers who add random filler to the “downtime” of a video, like giggles and animations on the screen, which seem to be using gimmicks to entrain visual attention in their videos (especially if the underlying content is simplistic/low-quality). Examples of a “1” code include:

- Extraneous animations and sound effects on top of live video in influencer videos [Baby doll and doctor baby Annabell doll - Kids pretend play with First aid kit for baby dolls & toys - YouTube](https://www.youtube.com/watch?v=a7aBWukgkzk)
- Cartoons that have extra visual enhancements not relevant to the story content such as stars, bubbles, visually-engaging/stimulating effects: [Dinosaur Candy Store | 🍬🦖 Do you have candy that I can headbutt? | Dinosaur Songs | JunyTony - YouTube](https://www.youtube.com/watch?v=gNuJewH8b6k)
- Giggling counts as bedazzling
- Pocoyo - has lots of extra sound effects with character movement that are not realistic and are very frequent.
- Lots of animations telling the viewer to like and subscribe (not just a little pop-up)

Example of a “0” code:

- Baby Einstein had images that were flashy/eye-catching, but slower pacing and not extraneous to the content of the video: <https://www.youtube.com/watch?v=_HbEejSqE9Y&ab_channel=BabyEinstein>
- When there are occasional visual and sound effects that match the action on the screen (e.g., the sound of a dog’s feet walking to the library) and/or call attention to a learning goal (e.g., a number 2 moving while a voice over says “two!”, sparkles around an object that is a learning goal), this is NOT bedazzling because it is not extraneous and would make sense to a young viewer watching the action on the screen.
- Examples of relevant enhancements that are a 0: Number blocks having sparkles/bedazzling around the number the child needs to pay attention to; a quick and simple transition screen

**Pos_role_modeling:**  This code describes when characters show discrete, observable pro-social or healthy behavior such as empathy, perspective taking, cooperation/helping, being flexible, caring for animals or sick people, calming down emotions, handling frustration, wearing sunscreen, eating healthy foods, brushing teeth, or safety; could include positive cultural or religious traditions). This can include having shown a negative behavior initially (which might be coded as **Neg_role_modeling**), but meaningfully resolving it (such as apologizing, trying to make things better). Positive Role Modeling and Negative Role Modeling are not mutually exclusive. This could also include song lyrics that are understandable to a young child (e.g., “Let it Go” from Frozen, but NOT the song we heard from Lord of the Rings) and include positive messages about emotions or health (e.g., “eat your vegetables”). How-to videos may act as positive role modeling if they show how to do something creative or resourceful; but will vary in level (1 or 2) based on how explicitly they are teaching children to do positive things. Simply being friendly or dancing together - without other expressions of prosocial behavior - does not count as positive role modeling.

**Examples of a “1”** are when clear and authentic prosocial behaviors are brief/discrete, OR are present to a low degree throughout the video. Based on social learning theory/informal learning, child viewers would absorb some positive role modeling just from watching if it is occurring throughout the video.

- Cocomelon or Elmo video where people are showing clear behaviors of being nice to one another, helping each other, saying please and thank you.
- Positive role modeling from other children doing something brave: [STRIDER Bikes Rule! 2 Year Old KTM Shredder - USA - YouTube](https://www.youtube.com/watch?v=Itsrgw17zQQ) or dance moves

**Examples of a “2”** (overt demonstration of positive behaviors/kindness - we think this would be more effective/memorable)

- Healthy eating habits [This Is The Way We Eat a Meal! | Sing Along with Hogi | Healthy Habits | Pinkfong & Hogi - YouTube](https://www.youtube.com/watch?v=7GTQiXZRppA)
- Little Bear being kind to friends throughout an entire episode - or any very strong role modeling of kindness
- Showing children how to do chores or activities of daily living
- Being a good friend; listening; being empathetic or understanding
- Storybots teaching about emotions
- Attention/empathy paid to different characters emotions or experiences
- Blippi teaching how to make holiday crafts that you can share with friends or family

**Examples of 0:**

- BRIEF, rare event of superficial kindness. Blippi saying please/thank you once or twice in a whole episode
- If doing something nice for someone is only the premise of the video so that they can show lavish things (e.g., decorating things extravagantly for someone’s bday), but there is not good role modeling of being caring for other people, then code a 0.
- Cry babies being surface friendly to one another

**Neg_role_modeling:** This code describes when any character in a video shows a negative, impulsive, violent, rude or unsafe behavior. Includes any unsafe behavior that a child might be tempted to do (even if a child wouldn’t be able to do it IRL). Even if the behavior might be appropriate for an adult to do (e.g., starting an engine), if it is performed by a child-directed character, or a character that treats the viewer like a “friend,” and there is no warning like “make sure a grown up does this” then it is negative role modeling. Videos can be coded as 1 for both positive and negative role modeling (i.e codes are not mutually exclusive). Videos may also be coded for negative role modeling if the characters over-focus on appearance, competition, or consumption.

**Examples of a “1”** include general observations of rude/bratty behavior or single omissions of safety rules (e.g., Blippi not saying “ask a grownup”):

- Taking something without asking (e.g., Blippi picking flowers from a public park) [Blippi's Favorite Vehicles in Real Life! | 2 HOURS of Blippi | Educational Videos for Kids - YouTube](https://www.youtube.com/watch?app=desktop&v=BnuDBHPmTdM&t=622s))
- Vanity (e.g., in an animal hair salon video)
- Brief unsafe behavior (e.g., Blippi dancing on a helicopter tarmac; cartoon child diving head first into a shallow pool during a swim lesson).
- Low-grade sassy/naughty or aggressive behavior that is recurrent or pervasive
- Influencers who focus on consumption/toys, but are not rude

**Examples of a “2” include:**

- Teasing, pranks, unsafe challenges, laughing at others who are hurt throughout the video: [Wheels On The Bus | Nursery Rhymes For Kids And Children - YouTube](https://www.youtube.com/watch?v=HI1VB1xTiS8)
- Any Foul language
- Any Physical violence (e.g., guns, hitting, car crashes)
- An influencer dreaming about doing naughty things or tricking their parents, even if they say sorry afterwards. [🎬 FULL SEASON 3 🎬 💧 CRY BABIES 💧 MAGIC TEARS 😍 EPISODES 💕 CARTOONS for KIDS in ENGLISH 🎥 LONG VIDEO - YouTube](https://www.youtube.com/watch?v=Bi_o6tST_KA)

**Counts as 0:**

- If there is a cranky or upset character whose behavior is part of a meaningful story, like Oscar the Grouch, but no other negative role modeling or negative consequences of that character’s behavior, code a “0”.
- If there is brief (<10 sec) risky or dangerous behavior shown and not role modeled or shown how to do by a main character

**Branded_content:** (0 vs 1) This code describes the central featuring or intentional placement of branded toys, food, candy, clothes, cars/vehicles, hotels, museums/destinations, etc. Character might say the brand name out loud (“here’s my lambo”), call explicit attention to the brand (“I’m using X brand markers”), or the video takes place in a store like Target, or is showing off a playspace or aquarium (e.g., Blippi). (If the characters in the video are branded, such as Mickey Mouse or Paw Patrol, code a 0 unless they play with or mention their own toys or other brands). Examples of a “1” include:

- Kid influencers who have placement of toys and brands in their video, even if they don’t name the brand. This could include:
  - getting deliveries of toys in the mail: [Diana and Toys Delivery - YouTube](https://www.youtube.com/watch?v=hxcPATllyKQ)
  - Having visible toys/branded products laying around their house, such as a Barbie pillow or Disney princess dress
- YouTubers unboxing a toy or performing reviews
- If the brand name is in the Title and is shown frequently (e.g., on construction vehicles)
- Kids playing with toys that happen to show certain brands such as McDonalds or NERF or Twix - need to say the brand name out loud OR feature the brand strongly in the storyline or frequent images in the video.
- Shopping videos
- In a DIY or tutorial video, YouTuber might specifically point out the brand name something they are using
- A brand’s own youtube channel: [STRIDER Bikes Rule! 2 Year Old KTM Shredder - USA - YouTube](https://www.youtube.com/watch?v=Itsrgw17zQQ)

**Counts as 0:**

- Unintentional brand placement, for example seeing truck brand names in a construction vehicle video or someone wearing a branded piece of clothing
- Brief showing of the name of a farm on its entry sign/fence
- In a movie clip, briefly showing the movie name/where to find it

**Vicarious_pleasure:** (0 vs 1) This code describes a video that has satisfying, hedonistic, or pleasurable content or animation - such as eating candy, playing with toys, making extravagant messes, showing off luxury items, wearing expensive outfits, or taking part in activities that kids might wish to do (e.g., go karts, having a birthday party, finding buried treasure, climbing on fire trucks; kids dancing with cartoon characters or watching a child doing something cool/fun). The purpose of the video may be to calm down/escapism, watch other people/characters do things the child may wish to do - so it is a vicarious experience. (in contrast to an interactive experience with call-and-response to “play” together, since that allows the child to be a participant).

Examples of a “1” include:

- Blippi going to aquarium or indoor playground
- Videos that just show a series of videos of construction vehicles, trains, tractors etc. [Fun With DIGGERS IN ACTION 🦺 Diggers At Work, Diggers For Kids | Excavator TV - YouTube](https://www.youtube.com/watch?v=B2IvnVp-FCI)
- Any sort of “Haul” video or segments in videos.
- Kids showing off their toy collections, candy they have, clothes they own.
- Videos in which there is a birthday party, cake, opening of presents
- Showing off their homes/cars/designer items, etc… (Vloggers living in a nice house is a 0, but if they make comments about their cool stuff or the views/beauty, that is a 1)
- Videos centered around kids playing with toys (Elaborate games set up with multiple toys, kids “pretend playing”) Allowing the viewer (child) to live through other children.
- Playing games that are easily won. (Plinko, finding items in a small tub of balls, spinning surprise wheels, congratulatory remarks for little to no effort)
- Watching another person play video games
- Watching fights
- Satisfying videos (“calm your baby down” animations, slime videos, illustrations, chocolate pouring, speed-building or painting)
- *Videos that show a picture-perfect family life where everyone is happy all the time* (e.g., Cocomelon) or extravagant adventure that a typical child would not be able to experience. Example: CocoMelon’s Beach Song depicting a happy, perfect day at the beach; Mickey Mouse Fun House Pirate Adventure.
- Dance videos that are in a pleasurable environment, such as Danny Go digging gems or appearing to be in a video game

Code a ‘0’:

- Videos in which the viewer is invited to come play/pretend with the characters.
- Lots of imaginative play and prompting the child to move their own body. The video can be themed and fun, but still center imagination
  - <https://www.youtube.com/watch?v=YR1OxBk8BF4>

**Educational_content (0/1/2).** This code describes the educational quality of videos, taking into account both the depth of curricular content and how it is delivered. For example, a video might have a lot of different labels or ideas in it, but if it is delivered in a fast pace that doesn’t tie it all together for the child (e.g., a Blippi video), it would be a 1. In order to code a 1 or 2, the coder needs to be able to identify a learning goal, such as preacademic skills (letters, numbers, shapes, colors), a healthy behavior, or social-emotional learning concepts. Positive role modeling may count as SEL in many videos. Just highlighting words on the screen (e.g., to sing-along with a nursery rhyme) is not educational. Repetition of song lyrics or reading a book without pointing to the words does not count as educational. NOTE: Some recent videos have had odd educational content that is either incorrect, seems poorly translated from another language, maybe AI generated, or seems to be geared towards grabbing a child’s attention (e.g., Baby Bus potty video). OK to downgrade the educational code of these videos.

Examples of a “1” include

- Video’s main point is entertainment or engaging kids, but has some surface educational content (e.g., numbers, colors, shapes, nature/animals, positive health or social behavior such as cleaning up) to be able to claim it is “educational,” but pays lip service to it.
- If there is a lot of fantastical content that could impose cognitive load (e.g., Mickey Mouse funhouse)
- Blippi pointing to leaves and naming their colors, but not explaining why leaves change colors or otherwise teaching about trees or colors.
- An ABC video that shows letter sounds/words but doesn’t go any farther
- Teaching or educational content is secondary to another main objective of the video (e.g., exploring tractors, unboxing toys): <https://www.youtube.com/watch?v=IPr_Ay-7aT0>
- Teaching about shapes (even if they show how where they appear around you) - when the rest of the content is fluff

Examples of a “2” include:

- Teaching approach explains a concept a few different ways relevant to kids, might tie it to children’s experiences, shows how knowledge could be applied in everyday life (e.g., breaks down how to brush teeth, why holiday traditions are meaningful; where to see shapes in everyday items)
- repeats the concept in a few different ways that all would help a child understand it (e.g., putting on sunscreen; fish in different places, doing different things, explaining how fish swim, etc).
- May have more educational teaching techniques such as pause-and-response.
- Has a narrative structure that has a story arc, not necessarily with a clear educational curriculum
- Video seems built around an intent to teach
  - Example: ["Emotions" - StoryBots Super Songs Episode 8 | Netflix Jr - YouTube](https://www.youtube.com/watch?v=akTRWJZMks0)
  - Ms Rachel speech pathology

**Notes to coders: What makes media educational for ages 18 mo to 5 years?**

**Great review chapter by Kirkorian et al:** [**https://drive.google.com/file/d/1_NVrpytbzS7qLZiOUWvJO4-sDDiDoclh/view?usp=sharing**](https://drive.google.com/file/d/1_NVrpytbzS7qLZiOUWvJO4-sDDiDoclh/view?usp=sharing)

- Relatively slower pace so that children can process the information - especially allowing call-and-response
- Lack of extraneous enhancements such as sound/visual effects or rapid switches or design features that are attention-grabbing. These impose more of a cognitive load and make it harder to learn the underlying concepts, or are distracting.
- Repetition, especially showing a concept in several different ways relevant to a child’s experience
- Using language and concepts that are simple and easy to understand
- Use of a character that the child knows and likes (e.g., Elmo, Ms =), since children pay more attention to them.
- Having a caregiver co-view and use media together (e.g., if the video explicitly suggests that a grown up watch/sing along)
- *Example: Nursery rhymes*
  - A more educational version of nursery rhymes would be sung by a character the child knows, allow for pause/call-and-response, shows sign language/gestures or different ways of understanding the words in the song (e.g., pictures of animals in Old McDonald)
  - A less educational version of nursery rhymes would be a cartoon that shows a bus picking up passengers while a soundtrack to “wheels on the bus” plays and words are shown along the bottom of the screen (which toddlers can’t read), + silly animations that appear on the screen that are not relevant to the song.
